# A scoping review of medical professionalism research published in the Chinese language

**DOI:** 10.1186/s12909-016-0818-7

**Published:** 2016-11-23

**Authors:** Xin Wang, Julie Shih, Fen-Ju Kuo, Ming-Jung Ho

**Affiliations:** Department of Medical Education & Bioethics, National Taiwan University College of Medicine, 1 Jen-Ai Road, Section 1, Taipei, Taiwan

**Keywords:** Medical professionalism, Scoping review, Chinese language

## Abstract

**Background:**

The Chinese Medical Doctors Association (CMDA) adopted the Charter of Medical Professionalism in the New Millennium (Charter) and published the Chinese Medical Doctor Declaration (Declaration). This is an important step to re-building medical professionalism in China at a time when the commercialization of health care has led to a decline in physician accountability and public trust in the profession. In response, authors have begun to examine and promote medical professionalism in China. This study aims to present the key research themes, identify research gaps and offer recommendations from reviewing the increasing pool of Chinese-language literature on medical professionalism.

**Methods:**

A scoping review of Chinese language papers was conducted using the China National Knowledge Infrastructure (including China Academic Journals Full-text Database, China Doctoral Dissertations Full-text Database, Masters’ Theses Full-text Database, China Core Newspapers Full-text Database, and China Yearbooks Full-text Database) (CNKI) database.

**Results:**

Four major research themes were identified in Chinese discourse: (1) teaching professionalism, (2) practicing professionalism, (3) conceptualizing professionalism and (4) assessing professionalism. Overall, authors were concerned with the cultivation of humanism in physicians and emphasized the importance of communication skills to improve the physician-patient relationship in China. They explored the role of traditional Chinese values, such as Confucian and Taoist values, as well as the Communist Party’s political values, in promoting professional behaviour.

**Conclusions:**

Authors demonstrate increasing interest in medical professionalism in China. The literature is of variable quality and further empirical studies are required in order to evaluate teaching interventions and guide professionalism assessment. A common professionalism framework is absent and could be developed with consideration to China’s socio-cultural context.

## Background

Medical professionals have made significant efforts to promote professionalism over the past two decades [1–4]. For example, the Physician Charter, published in 2002 by American and European medical associations, affirms that physicians should place patient welfare above the pursuit of self-interest [5]. Past reviews of professionalism literature have examined research trends and educational practices [6–16]; however, these reviews have focused only on English-language publications. In a global age, there is value in examining medical professionalism from an international perspective, particularly given the increasing recognition of cultural differences in the construct of professionalism [2, 17–20].

China in particular has increased its contributions to professionalism literature as growing attention has been paid to medical education and medical professionalism in China [21–23]. The Chinese Medical Doctors Association (CMDA) adopted the Charter of Medical Professionalism in the New Millennium (Charter) in 2005, and drafted the Chinese Medical Doctor Declaration (Declaration) six years later. The Declaration not only takes Chinese traditions into account, but also considers current social circumstances by emphasizing the virtue of incorruptibility and integrity [24]. This is an important step to re-establishing medical professionalism in practice at a time when physicians in China are influenced by financial interests (e.g. over-prescription which is rooted in the fee-for- service payment system and distorts the regulation of price) [24]. Meanwhile, several Chinese research organizations on physician professionalism were established, including the China-U.S Center on Medical Professionalism of Peking University Health Science Center and the Center for Research on Medical Professionalism of CMDA. A series of professionalism seminars have been held since 2006 including five China-U.S. Conferences on medical professionalism.

Consequently, authors have begun to recognize the importance of promoting medical professionalism in China. Hundreds of Chinese-language papers have been published on medical professionalism in recent years, examining topics from physician-patient relations to medical curriculum design. While there have been reviews of Chinese-language medical education literature [25], no studies to date have specifically examined trends in medical professionalism. We believe it is important to understand medical professionalism in China not only because of the volume of physicians, researchers and medical educators produced there, but also for other countries like India, Pakistan and Bangladesh where many doctors receive their medical training in China [26–28]. In order to understand the scope of Chinese medical professionalism research, this study reviews and summarizes Chinese literature on medical professionalism, and offers recommendations for future research.

## Methods

A scoping review approach was conducted for this study. The scoping review is a strategy designed to provide conceptual clarity about a specific topic or field of literature through the synthesis and analysis of a wider range of literature. In comparison with systematic literature reviews, scoping reviews do not typically assess the quality of included material. Instead, they typically focus on the breadth of the selected studies by ‘mapping’ existing literature in a certain field and identifying research gaps [29, 30]. This approach is particularly helpful for complicated topics and topics that have not been systematically reviewed before[30]. We adopted the Arksey and O'Malley’s methodology framework of scoping review approach[29], which comprises of five stages: (1) identifying the research questions, (2) identifying relevant studies, (3) study selection, (4) charting the data and (5) summarizing and reporting results.

### Identifying the research question

The research question for this review was developed by the authors and was outlined as: What research questions/topics/areas have been studied in medical professionalism research in Chinese language literature?

### Identifying relevant studies

China National Knowledge Infrastructure (including The China Academic Journals Full-text Database, China Doctoral Dissertations Full-text Database, Masters’ Theses Full-text Database, China Core Newspapers Full-text Database, and China Yearbooks Full-text Database) (CNKI) was selected for being the most comprehensive and widely used Chinese journals database. CNKI includes journals published in China and excludes journal publications from Hong Kong and Taiwan. A search was conducted for articles on Chinese medical professionalism in CNKI’s Medicine and Public Health series published between January 1994 and December 2014. Chinese terms related to medical professionalism were used as keywords, including “职业精神” (*zhi ye jing shen*, professional spirit), “专业素养” (*zhuan ye su yang*, professionalism) and “人文精神” (*ren wen jing shen*, humanism). We selected articles that present an issue or situation of China, including works from non-Chinese authors who collected their data in China and published in Chinese. Note that studies focused on traditional Chinese medicine and other health care professionals (HCP) rather than on physicians and medical students are not included in this research as they might have a different code of professionalism from that of medical professions.

### Study selection

**Fig. 1:**
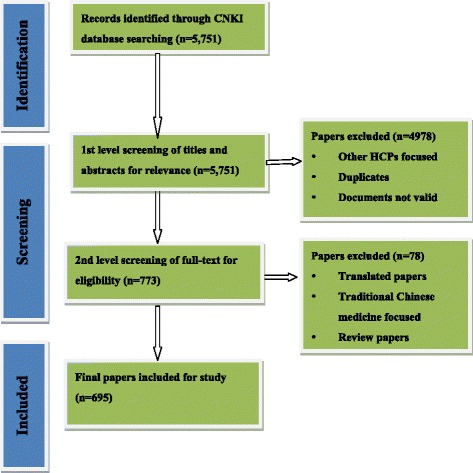
Overall flow of scoping literature search and selection

Articles directly matching or approximating these keywords were identified. Titles and abstracts of articles were filtered for relevance to medical professionalism and the full content was examined to determine suitability for inclusion in the review. We included all peer reviewed papers that addressed any aspect of medical professionalism. The search covered 1994–2014. An initial screening of 5751 titles and abstracts was undertaken. Duplicates and any papers not meeting a broad inclusion/exclusion criterion were discarded. Seven hundred seventy-three papers went through to a second level of screening of relevance. A further 78 papers were excluded, including articles translated from other languages; studies focused on traditional Chinese medicine; and studies that focus on other health care professionals (HCP) rather than physicians and medical students (see Fig. 1).

### Charting the data

In line with Levac’s approach [30], we adopted a preliminary step for data charting. Two researchers independently reviewed a random sample of 10 articles, then drafted and tested the data abstraction form to make sure their approach to data extraction is consistent with the research question. The final form included two general headings: study characteristics (e.g., year of publication) and research topic area. The coding process included three main phases: open coding, creating categories and abstraction [31]. Open coding involved three researchers independently reading and writing notes and headings to describe the main research area for each article. The heading and notes were then recorded to generate a list of initial codes. The list was then refined through a cyclical process by combining similar codes into subcategories. During the abstraction phase, based on semantic and conceptual similarity, subcategories were condensed into four major themes. Debriefing meetings were conducted to confirm interpretations, coding decisions and the development of categories. Coding was conducted using qualitative data analysis software NVivo [32].

### Collating, summarizing and reporting results

The data were synthesized according to topic areas of medical professionalism. Data analysis mainly involved qualitative thematic analysis. The results are presented below in figures and narrative forms.

## Results

Of the 5751 studies initially identified, 695 articles met the authors’ criteria for inclusion in this review. These covered a variety of topic areas associated with medical professionalism. Thematic analysis was conducted on these selected articles and four main research areas were identified.

**Fig. 2:**
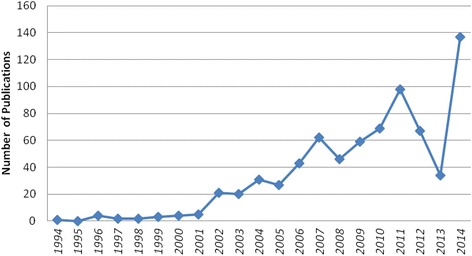
Number of publications on professionalism in the CNKI database from 1994 to 2014

**Fig. 3:**
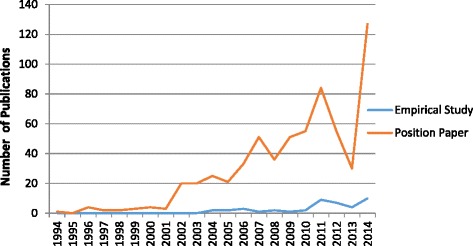
Number of publications on professionalism in the CNKI database: Empirical studies VS. position papers

**Fig. 4:**
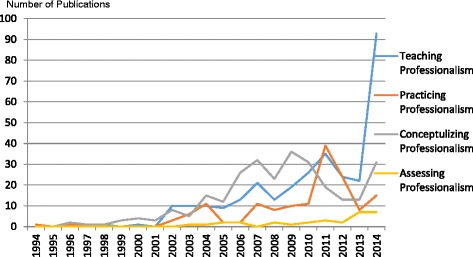
Number of publications on professionalism in the CNKI database for each theme

**Fig. 5:**
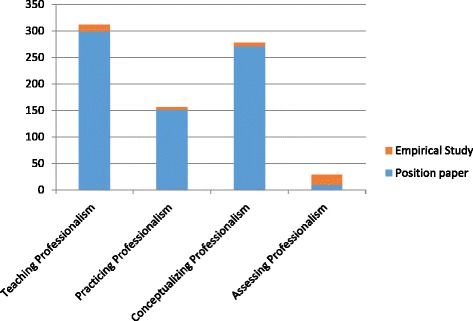
Number of empirical studies and position papers in the CNKI database for each theme

A distinct rise in the quantity of medical professionalism literature was observed starting in 2002 (see Fig. 2). Of the 695 articles selected in this review, only 42 of the articles were published prior to 2002; the remaining 653 articles were published in the subsequent decade. The content of medical professionalism literature also changed over time. Before 2002, research focused on the importance of humanism in medicine and the integration of science and humanism. After 2002, articles place more attention on current challenges in medical professionalism such as the deterioration of the physician-patient relationship. Of the literature reviewed, it is important to note that the majority of articles were position papers, with few empirical studies conducted. Of the 695 articles selected in this review, only 43 of them were empirical studies; the remaining 652 articles were positioning papers (see Fig. 3). Four main themes were identified: teaching professionalism, practicing professionalism, conceptualizing professionalism and assessing professionalism in China’s cultural context (see Figs. 4 and 5). Note that a single article may address more than one theme.

### Research themes

#### Teaching professionalism

Many of the reviewed publications emphasize the importance of teaching professionalism in medical schools and residency training programs in order to foster the professional growth of young physicians [33, 34] and cultivate their humanistic knowledge [35, 36]. In China, it is considered a teacher’s responsibility to help medical students cultivate patient-centered values [37]. However, He and colleagues [38] note an over-emphasis on core sciences in medical curricula, as social sciences are taught separately from medical sciences in China. Few humanities courses are offered and, when available, they are often optional electives [36, 39]. Despite an interest in integrating social sciences into medical education, authors also express concern with the scarcity of humanities teachers, poor teaching standards and the need for continuing education programs to train educators [36, 40–42].

Authors suggest several areas for improvement in medical education, including new teaching models and methods to cultivate professionalism. Si [36] calls for a more engaged teaching style and a move away from lecture-based courses, suggesting that group discussions be used to elicit participation and monitor improvement and understanding among students. Other authors note the importance of role-modeling, teacher behavior and school environment for the cultivation of professionalism [39, 40]. In order to develop critical thinking skills and humanism, some authors encourage learning experiences outside of the classroom, such as extra-curricular activities or volunteer work in the community [36, 42]. Overall, authors express a pressing need to integrate professionalism into medical education through greater availability of humanities courses and participatory learning experiences.

#### Practicing professionalism

The second major theme discussed in the literature relates to current challenges in practicing professionalism. Medical schools and resident training programs in China are striving for professional growth in young physicians and recent economic and social changes have drawn renewed attention to the importance of professionalism. A central challenge to practicing professionalism is the commercialization of China’s health care system, which has produced financial incentives that distort professional ethics, damage public trust in the profession, and compromise the physician-patient relationship [43]. One study found that Chinese patients expressed far more dissatisfaction with the irresponsibility of physicians (44.8%) than with physicians’ medical techniques (14.5%) [44]. Patients cited physician irresponsibility, medical errors and high medical expenses among their concerns, and 39.2% of patients believed that physicians prioritized profits over patient welfare [44].

Other articles examined the cause of damaged physician-patient relationships. For example, Liu [45] highlights a central flaw in the Chinese medical system in which physicians’ salaries are directly connected to their treatment decisions, leading professionals to order expensive treatments or overprescribe. Zhao [46] discusses the problematic practice of patients offering red envelopes of money to physicians in order to receive better services. Other studies express concern that the absence of a comprehensive self-evaluation system worsens physician-patient relationships [47]. Meanwhile, editorial bias in mass media was criticized for damaging public trust in the profession as news about doctor-patient disputes were deliberately over-hyped to attract viewers and readers [48].

Apart from economic and social changes, new developments in medical practice hinder communication and professional accountability. Widespread adoption of technology results in physicians focusing on technical diagnoses rather than communication with their patients [49]. Research demonstrates that health providers perceive themselves as lacking in humanism [50], especially as current physicians attend to large numbers of patients and experience fatigue [51]. Lack of professional guidelines added to the pressures of a heavy workload and time constraints experienced by physicians can hinder proper patient care [52]. In Chinese discourse, improved communication is repeatedly emphasized as crucial to mending physician-patient relations [49].

Overall, authors recognize that a spirit of medical humanism is foundational to the physician-patient relationship and are concerned with rebuilding professional values in order to regain the public’s confidence [45, 53, 54]. They attribute the deterioration of the physician-patient relationship to distorted financial incentives, media sensationalism and lack of communication.

#### Conceptualizing professionalism in China’s cultural context

In order to address the current deficiencies in medical professionalism, many authors examine and promote professionalism in the context of traditional Confucian and Taoist values [55], while others focus on contemporary Chinese Communist Party’s political values and their influence on professionalism development.

The philosophies of traditional medicine are influenced by Confucian and Taoist thought, and some authors argue that the current deterioration of medical ethics may be addressed by reclaiming the moral foundations of Chinese medicine that promote respect for life, nature and other individuals [50]. For example, a traditional Chinese value emphasized by some articles is “医乃仁术” (*yi nai ren shu*, benevolent medical practice), a Confucian concept calling on physicians to treat patients fairly regardless of their wealth. Other Confucian values, “仁者” (*ren zhe*, benevolent person) and “修身” (*xiu shen,* self-discipline), promote moral accountability and encourages physicians to actively seek improvement and overcome deficiencies [56, 57]. Authors discuss the concept of “和” (*he*, harmony) to repair the physician-patient relationship. The phrase “阴阳调和、医患信和” (*yin yang tiao he, yi huan xin he*, harmony of opposites, harmony of physician and patient) refers to the Taoist belief that the physician-patient relationship must be balanced by mutual trust and respect, in which physicians prioritize patient welfare over personal gain [40]. Authors call upon modern physicians to incorporate these traditional values into their professional ethics, and in doing so, reclaim the dignity of their profession while constructing a contextualized definition of professionalism that grounds itself in China’s cultural tradition and values.

The Chinese Communist Party's political values are also discussed in relation to professionalism development. The socialist core values proposed in the Communiqué of the Sixth Plenum of the 16th Central Committee of the Communist Party of China (CPC) in 2006 are regarded as the essence of socialist ideology and promoted by the CPC nationally. Authors suggest that these core values can be used to facilitate medical professional ethics development. Li and Xu [58] suggest that the principles of medical professional ethics (autonomy, beneficence, nonmaleficence and justice) are in line with and reflected by the socialist core values, which include freedom, equality, justice, the rule of law, dedication, integrity and friendship. They could therefore be integrated to create a unique framework of professionalism that suits contemporary China [58, 59]. Similarly, the Communism Bethune spirit is also analyzed in the literature as a philosophy developed during World War II emphasizing reverence for life, loyalty to patients, professional ethics and dedication [60]. Authors argue that the values of Bethune spirit are consistent with the Geneva Declaration and could assist the development of professionalism in China [60–62].

In addition to the examination of traditional and political values, there have been efforts to define a comprehensive professionalism framework that China currently lacks. Li and Wang [63] evaluate domestic studies and consider traditional Chinese cultural values to propose seven principles of Chinese professionalism: altruism, humanism, devotion, sacrifice, creativity, teamwork and critical thinking. According to Li and Wang, Chinese medical professionalism is distinguished not only by basic medical humanism, but also by moral obligation and creative character. The literature demonstrates Chinese authors’ attempt to promote professionalism by aligning professional values with existing philosophies grounded in China’s history and cultural context.

#### Assessing medical professionalism

The fourth major topic identified focuses on medical professionalism assessment in China. Assessment-themed papers are generally review articles rather than original research articles and echo internationally known literature in recognizing professionalism assessment as the basis for enhancing medical professionalism and advocating a critical approach to select assessment tools based on content validity, reliability and impartiality [12, 15]. For example, Yang and colleagues [64] argue that improving professionalism assessment is a core competency for professionalism development and the existing assessment system in China needs to be more comprehensive. Yang and colleagues [64] reviewed existing assessment methods used in western countries in terms of ethical knowledge and reasoning. Assessment tools, such as the global performance rating, professionalism mini-evaluation exercise, defining issues test (DIT) and 360-degree evaluation, were analyzed in detail to determine their applicability to the Chinese context. Based on their review, 360-degree evaluation was determined to be the most applicable method for resident assessment and DIT a better method for medical students with limited working experience [64].

Chen and colleagues [65] performed a similar review examining the four assessment methods (360° evaluation, objective structured clinical examination, professionalism mini-evaluation exercise and conscientiousness index) based on their target assessment group, types of outcomes assessed and how they could be adopted into the Chinese context. Consistent with Chen and colleague’s recommendations [65], Chen and Cao [66] suggested that assessment tools in China should be developed using widely applied methods. Their literature review of professionalism assessment in Chinese-language databases found that primary research on professionalism assessment was scarce and the scope of the questionnaires did not cover all the key elements of the Declaration of Geneva. The authors conclude that compared to western countries, professionalism assessment in China is still at an early stage of development and there is a pressing need to develop a unified and reliable assessment method [66]. Authors call upon domestic researchers and medical educators to improve the existing assessment system by studying and tailoring existing well-developed methods instead of developing a new one, a process both resource-intensive and unnecessary as shown by previous literature [10].

## Discussion

This scoping review of medical professionalism literature in Chinese language provides important insights into the field of medical professionalism. It observed an increase in the number and variety of papers on this topic over the past two decades. In the years following the release of the Physician Charter in 2002, the number of publications on CNKI increased, reflecting a rising concern with improving the state of medical professionalism and perhaps signaling the Charter’s influence on international professionalism dialogue. Authors are actively debating areas for improvement in the teaching and practice of medical professionalism. In particular, they emphasize cultivating humanism among physicians and medical students in China. Improved communication is also considered crucial to rebuilding trust in the physician-patient relationship, which has deteriorated as a result of distorted incentives and technological advances in the current health care market.

This study also reveals the influence of culture on professionalism. Some authors analyze professionalism through the lens of traditional Confucian and Taoist values, supporting the idea of professionalism as a context-sensitive concept [18, 20, 67]. Ancient cultural values emphasizing the practice of humanistic medicine may be used to address China’s crisis in professionalism. Chinese physicians could assert their own context-specific professional identity by establishing a professionalism framework that reflects cultural heritage [68], as has already been achieved at one Chinese medical school [69] and is reflected in experiences in the Middle East [17, 70]. Medical professionalism curricula can also be developed with consideration to local culture and needs, as has been demonstrated in Taiwan and Japan [71, 72]. Despite the modernization of medicine in China, traditional culture continues to influence perceptions of professionalism and societal expectations of physicians [69, 73]. Appropriate cultural considerations should be made as China continues to promote medical professionalism,

Political influences on professionalism are also identified in this review. Authors analyze professionalism development in light of the political values promoted by the Communist Party of China (CPC), comparing them with medical professional ethics and the Geneva Declaration. The authors suggest that some of the CPC’s political values could be used to facilitate professionalism development in China as they share similar ideas with the principles of professionalism identified by authorities from the West [58, 60, 62]. We learn from the review that there exists a unique political influence on medical professionalism in China. When staying politically neutral is difficult, authors respond to political pressure by trying to achieve a balance between professional autonomy and political acquiescence through careful negotiation of the officially promoted political values. Researchers should bear in mind this ubiquitous influence in their future work.

This scoping review also reveals research gaps in Chinese language literature. While Western discourse has made strides towards defining measurable traits of professionalism [2, 16], there are few in-depth studies defining and assessing professionalism in Chinese-language literature, though some Chinese assessment studies have been published in English [74, 75]. Furthermore, a common definition of professionalism continues to be debated in international academic literature [76, 77], a challenge that extends to the Chinese context. It could therefore be beneficial to establish a working framework of professionalism in which to ground measures of professional attributes and from which to gauge future improvement.

Although medical professionalism education is identified as a priority, there has been little examination of current interventions and their effectiveness. Evidence that communication skills training and integrated learning approaches are effective in the Chinese context can be built upon through the development of training strategies, adoption of student-centered teaching methods and validation of assessment instruments [78]. There is also a lack of studies giving voices to those who have had personal experiences with professional dilemmas. Gaining greater insight into medical students’ narratives and perceptions of professionalism may prove valuable in identifying and addressing deficiencies [8, 79–82].

In the West, a focus on self-reflection and mindfulness has ushered in the use of narrative medicine, service learning and portfolio learning [83], methods not widespread in China despite a similar push to integrate humanities into medicine [84]. Though some Chinese medical schools have begun to adopt innovative teaching methods such as problem-based learning, resource constraints and the entrenchment of traditional teaching methods hinder widespread adoption of these approaches [84, 85]. Role-modeling and environmental influences are also identified as important aspects of professional development in Chinese medical schools, and the influences of the hidden curriculum on professionalism are worth further investigation [86].

Overall, there is a need for future studies to assess and improve the state of medical professionalism in China. Medical curriculum reform will continue to spread as all medical schools are urged to change their curriculum to integrate professionalism and to develop applicable assessment methods [87]. Long-term planning and management of medical schools will be necessary to introduce humanities into medical education and to encourage the cultivation of humanism [36, 40–42]. Government-specified standards should be developed for China’s existing 3 year medical education program that provide qualified general practitioners for rural villages as well as the new 8-year program [88]; funding and support should be continually provided to medical schools to facilitate their development of new teaching methodologies and assessment tools [85, 89, 90].

We identify some limitations in this review. Although the articles included were academic publications listed in CNKI, the majority were position papers rather than empirical studies. Thus, there remains little empirical evidence informing the state of medical professionalism in China, making it difficult to draw a conclusive or comprehensive picture of the situation. However, it is possible that other studies of professionalism in China exist, but were unpublished or published in other languages and consequently not covered in this review. Nevertheless, the aforementioned gap in the literature and the prevalence of non-empirical papers on the topic demonstrate the need for further research in the field of Chinese medical education, as previously called for by other scholars [25].

Cultivating the professionalism of medical students in China will have far reaching implications for the future of medical practice, particularly in terms of prioritizing patient welfare [91]. It is worth noting that China’s medical schools are currently accepting large numbers of students from other parts of Asia, such as India, Pakistan and Bangladesh [26, 27]. The internationalization of Chinese medical schools has facilitated student exchanges [92] and led to a greater number of Chinese-educated physicians practicing abroad [93], carrying with them values instilled during their education in China that may conflict with cultural and social constructs in other parts of the world [94]. With the globalization of medical education giving rise to international medical graduates [95], it is imperative that the next generation of physicians educated in China is professionally accountable.

## Conclusion

This scoping review of medical professionalism literature in Chinese language demonstrates a growing concern with cultivating humanism and integrating professionalism into both medical curricula and practice. Authors are interested in improving education systems and patient-physician relationships, with an emphasis on improved communication skills, rigorous assessment methods and the prioritization of patient welfare above self-interest. There has also been a re-examination of traditional Confucian and Taoist values of compassion and self-discipline as well as contemporary political values as China’s physicians establish their own local professional identity. Continuing efforts to teach, practice and assess professionalism are necessary in order for China’s medical profession to cultivate humanistic practitioners and regain public trust.

## Abbreviations

CNKI: China National Knowledge Infrastructure
CPC: Communist Party of China
DIT: Defining issues test
HCP: Health care professionals
